# Deep brain stimulation for myoclonus-dystonia syndrome with double mutations in DYT1 and DYT11

**DOI:** 10.1038/srep41042

**Published:** 2017-01-19

**Authors:** Jia-Wei Wang, Ji-Ping Li, Yun-Peng Wang, Xiao-Hua Zhang, Yu-Qing Zhang

**Affiliations:** 1 Beijing Institute of Functional Neurosurgery, Department of Functional Neurosurgery, Xuanwu Hospital, Capital Medical University, P.R. China

## Abstract

Myoclonus-dystonia syndrome (MDS) is a rare autosomal dominant inherited disorder characterized by the presentation of both myoclonic jerks and dystonia. Evidence is emerging that deep brain stimulation (DBS) may be a promising treatment for MDS. However, there are no studies reporting the effects of DBS on MDS with double mutations in DYT1 and DYT11. Two refractory MDS patients with double mutations were treated between 2011 and 2015 in our center. Genetic testing for DYT1 and DYT11 was performed through polymerase chain reaction amplification and direct sequencing of the specific exons of genes. For the first patient, initial bilateral ventral intermediate thalamus nucleus (Vim) DBS was performed. Because of worsening dystonia after initial improvement in symptoms, subsequent bilateral globus pallidus internus (GPi) DBS was offered at 43 months after initial surgery, which reversed the deterioration and restored the motor function. For the second patient, initial improvement in motor symptoms and quality of life was sustained at the follow-up 6 months after bilateral Vim DBS treatment. Thus, DBS may be an effective therapeutic option for MDS, even in patients with double mutations. Moreover, GPi DBS may be used as a supplementary treatment when initial Vim DBS fails to control MDS symptoms.

As an autosomal dominant inherited disorder characterized by the presentation of both myoclonic jerks and dystonia, myoclonus-dystonia syndrome (MDS) has received considerable attention in clinical practice^1^. MDS is most commonly caused by the mutation in the epsilon-sarcoglycan gene (SGCE, DYT-11) on human chromosome 7q21^2^,^3^, with its onset in childhood and early adolescence. Although MDS tends to follow a more benign course in most cases, the abnormal movements can significantly impair daily life activities in a subset of patients^1^,^4^. Unfortunately, although deleterious effects of MDS have long been recognized, medical and surgical interventions remain limited, and advancements in treatment have been relatively non-existent.

Current mainstay for the management of MDS consists of pharmaceutical treatment and surgical intervention. A broad collection of medications has been tried to treat MDS, including benzodiazepines, anticholinergics, dopaminergics, antiepileptics, serotonergics and neuroleptics^1^,^5^. However, in general, the response to these drugs is usually poor or even absent in the vast majority of MDS patients. It is well-known that deep brain stimulation (DBS) has been recognized as an efficient therapeutic strategy in several movement disorders such as Parkinson’s disease^6^, essential tremor^7^ or dystonia^8^. Based on the experience with DBS in abovementioned disorders, new evidence is emerging that DBS may be also useful in the treatment of MDS patients^9^,^10^. To our knowledge, there are forty-eight MDS patients treated with DBS that have been reported in the previous literature, which has demonstrated a trend for significant clinical benefit in improving myoclonic and dystonic symptoms after neurostimulation. The DBS targets involve the globus pallidus internus (GPi) in 35 cases^4^,^11^,^12^,^13^,^14^,^15^,^16^,^17^,^18^,^19^,^20^,^21^,^22^,^23^,^24^,^25^,^26^,^27^,^28^, ventral intermediate thalamic nucleus (Vim) in 4 cases^28^,^29^,^30^,^31^, and both GPi and Vim in 9 cases^28^,^32^ respectively. However, the optimal target remains unclear because the rarity of the disease limits access to mount randomized trials addressing the benefits of DBS. In addition, to date, the information about the effects of DBS on pediatric MDS patients is pretty insufficient. Moreover, to our knowledge, there are no studies reporting the effects of DBS on MDS with double mutations in DYT1 and DYT11.

To add to the scarce literature, we, therefore, reported two refractory MDS patients with double mutations in DYT1 and DYT11 treated with bilateral Vim-DBS or GPi-DBS. Our aim was to evaluate its efficacy in ameliorating abnormal involuntary movements and improving quality of life in these patients.

## Results

As shown in Table 1, the mean age of the two MDS patients at surgery was 23.5 years old while the mean age of onset was 11.5 years old. The mean latency to surgery was 12 years. Genetic testing indicated double mutations in DYT1 and DYT11 in the two patients. The predominant symptoms on admission involved significant myoclonus and hand dystonia. The specific distributions of myoclonus and dystonia were listed in Table 1. In addition, the symptoms of MDS in P1 were alcohol-responsive while the alcohol sensitivity in P2 was not tested because he was less than 18 years old. The mean duration of follow-up was 30 months.

**Figure 1:**
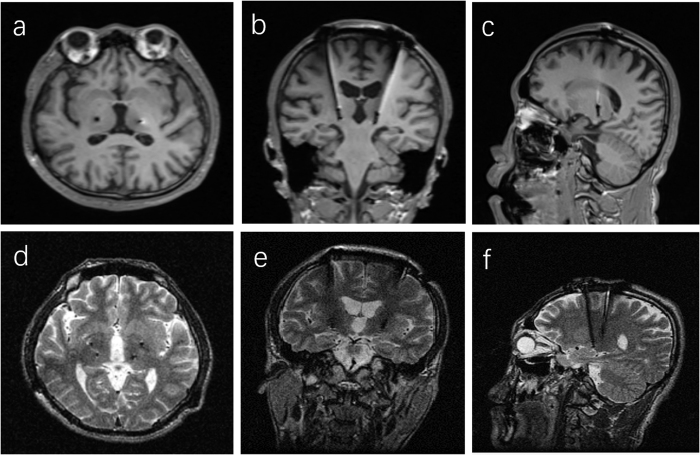
Representative imaging of DBS electrode localization in the MDS patient P1. (**a–c**) Postoperative T1-weighted MR images after initial Vim DBS. (**d**–**f**) Postoperative T2-weighted MR images after subsequent GPi DBS. **a/d**: axial plane, **b/e**: coronal plane; **c/f**: sagittal plane.

DBS surgery was provided to the two patients. Post-operative MRI or CT scanning was performed at the first day after surgery and then was co-registered with pre-operative MRI images through StealthStation^®^ surgical navigation system (Medtronic, Minneapolis, USA) to confirm the final position of the lead tips (Table 2). As for the patient P1(Fig. 1), bilateral Vim DBS was initially performed with improvement in MDS symptoms and quality of life. However, worsening dystonia occurred at 30 months after initial Vim DBS. Thus, subsequent bilateral GPi DBS was offered at 43 months after initial surgery when Burke-Fahn-Marsden Dystonia Rating Scale (BFMDRS) movement subscore deteriorated to 42 and BFMDRS disability subscore to 19 at 43 months after initial surgery. Supplementary GPi DBS reversed the deterioration and restored motor function. As for patient P2, the improvements in motor symptoms and quality of life persisted at the follow-up 6 months after single bilateral Vim DBS treatment. In total, the BFMDRS movement subscore decreased from 21 in P1 and 35 in P2 preoperatively to 3.5 and 8 postoperatively with the BFMDRS disability subscore from 16 in P1 and 17 in P2 preoperatively to 4 and 5 postoperatively. And the Unified Myoclonus Rating Scale (UMRS) rest/action subscore decreased from 36 in P1 and 24 in P2 preoperatively to 1 and 0 postoperatively. Overall, the mean improvement was 80.2% for dystonia and 98.6% for myoclonus at the last follow-up.

## Discussion

To the best of our knowledge, this study reported for the first time the efficacy of thalamic or pallidal neurostimulation for myoclonus-dystonia syndrome with double mutations in DYT1 and DYT11.

The clinical manifestation of MDS in this case series occurs at a mean age of 11.5 years old, which is consistent with previous reports indicating the onset of disease commonly occurs at the first or second decade of life^1^. Generally, the clinical phenotype of MDS is heterogeneous with variable expression of myoclonus and dystonia. As the most commonly clinical feature of MDS, myoclonus usually affects head, neck, and upper limbs, but occasionally extends to lower limbs. Dystonia, usually manifested as neck dystonia or hand dystonia, often coexists with myoclonus and may occasionally be the only symptom of the disease. In addition, psychiatric symptoms, including depression, anxiety, and obsessive-compulsive disorder, have also been reported in some families. The clinical symptoms of MDS with double mutations in DYT1 and DYT11 are unclear because of the rarity of the disease itself. Moreover, DYT1 is usually not detected in MDS patients with DYT11 mutation. A previous study reporting two individuals with double mutations has demonstrated that the presenting symptom is myoclonus while dystonic symptom occurs later^33^.

Torsin A (DYT1 gene) belongs to the superfamily of ATPase chaperone-like proteins that are involved in protein trafficking, refolding, and degradation^34^. The DYT1 dystonia is caused by the GAG deletion in the coding region of the Torsin A gene. Mutant Torsin A may lead to abnormal neurotransmission and disturb the neuronal firing in the motor pathways in the brain^35^,^36^. SGCE (DYT11 gene) is a part of the dystrophin-associated glycoprotein, which is distributed in midbrain neurons, cerebellar Purkinje cells, the hippocampus, and cortex^37^. Mutation in SGCE seems to lead to its mis-localization from the plasma membrane to the endoplasmic reticulum and the promotion of its degradation by the proteasome^38^. To date, little is known about the interaction between the DYT1 mutation and DYT11 mutation. *In vitro* data have indicated torsin A, that is mutated in DYT1 dystonia, binds to and promotes the degradation of SGCE mutants (DYT11) when both proteins are co-expressed in transfected cells^38^. Therefore, it can be speculated that loss or reduction of the torsin A function (e.g., occurring in DYT1 dystonia) may affect the quality control of SGCE and alter the amount of SGCE *in vitro*. Moreover, an animal experiment has demonstrated simultaneous mutations in two dystonia genes (DYT1 and DYT11) facilitate the onset of motor deficits in mice, suggesting that the additional multiple mutations are risk factors of early onset in this disease^39^. Further research on MDS patients with double mutations may provide clinical insight on this issue.

Previous studies have shown that there is a trend toward improvement in motor symptoms in patients with younger age at surgery and with a shorter duration between age at diagnosis and age at surgery, either in dystonia^40^ or in MDS^9^,^10^. Thus, DBS surgery was offered to our patients, including the individual (P2) who was only seventeen years old. To our knowledge, there are only four MDS patients younger than 18 years old at surgery with the age ranging from eight to seventeen years old who have been reported in previous literature^4^,^17^,^22^,^23^. For example, Cif and co-authors found there was an improvement of 92% in UMRS rest/action subscore and 86% in BFMDRS total score in the eight-year-old MDS patient at 20 months after GPi DBS^4^. In addition, an improvement of 57.1% in BFMDRS movement subscore and 31.3% in UMRS scores (items 2–5) was found in a fourteen-year-old MDS patient 16 weeks after GPi DBS^23^. Moreover, Kuhn and colleagues also presented a case of early successful treatment of MDS by GPi DBS in a patient at the age of 17 years leading to 83% reduction in dystonia score and 89% reduction in myoclonus at 9 months after surgery^17^. In our case series, the duration of follow-up in the young individual is six months (P2). The long-term benefit of the DBS surgery in this specific population is yet to be demonstrated.

Although the optimal stimulation target for deep brain stimulation in MDS is unclear, emerging evidence from clinical and basic studies have indicated the GPi or Vim may be the potential targets for DBS in the management of MDS. For example, a previous study exploring the local field potential recordings of the GPi from two genetically proven MDS patients has shown that the low-frequency band (3 to 15 Hz synchronization) may point to the existence of myoclonus-dystonia specific oscillatory activity in the GPi^14^. Moreover, in another MDS patient, electrophysiological data have also indicated the phasic pallidal activity correlates with and leads the myoclonic muscle activity, and the myoclonus is suppressed by bilateral pallidal DBS^19^. In addition, a functional magnetic resonance imaging study in a pediatric MDS patient has demonstrated specific abnormal activation in subcortical structures during action, including the thalamus and the dentate nucleus^41^. A [(18)F]-fluorodeoxyglucose PET study has also found significant DYT11 genotype-specific metabolic increases in the thalamus^42^. These data have suggested that the GPi or Vim may be involved in the pathophysiology of myoclonic dystonia.

Generally, the GPi has been well-accepted as the first choice for the DBS treatment of generalized and segmental dystonia while the Vim is the one for the essential tremor and Parkinson’s disease tremor^7^,^40^. Moreover, a wealth of evidence has indicated that thalamic DBS is also a feasible and effective therapeutic option for dystonia patients, including writer’s cramp^43^,^44^,^45^, dystonia tremor with a mild dystonia^46^,^47^, and DYT6 dystonia^48^. Based on these data, in this case series, Vim DBS was offered to the two patients with predominant myoclonus (P1) or hand dystonia (P2) as the chief complaints. Our results have indicated Vim DBS may improve myoclonus and hand dystonia, which is consistent with the previous study^10^. It is noted that one patient in this series (P1) presented with a worsening of dystonia 30 months after initial Vim DBS surgery although the efficacy of suppressing myoclonus was maintained. Worsening of dystonia has also been reported as a stimulation-induced adverse event following Vim DBS in the largest group of MDS patients with GPi and Vim DBS treatment^28^. The reason underlining the worsening dystonia is unclear, and may be associated with the disease progression, especially in the MDS patients with double mutations (DYT1 and DYT11). Fortunately, worsening dystonia in this patient was improved by subsequent GPi DBS, suggesting further GPi-DBS may be useful in cases of incapacitating or worsening dystonia, refractory to previous Vim-DBS.

It should be noted that there are several limitations in the present study. First, MDS is an autosomal dominant disorder with maternal imprinting. It is important to depict the detailed patient pedigrees in the setting of monogenic disorders, especially when the unusual double mutations coexist. However, the results of the motor examination, genetic analysis, psychiatric evaluation, and neuropsychological testing for the individuals in the patients’ family are absent in the present study. We have realized this issue and have begun to explore the patient pedigrees in our center. Second, because there are only two MDS patients treated with DBS in the present series, the effects of Vim DBS and GPi DBS on MDS are reported individually for each patient instead of being compared after pooling for each target. Thus, we cannot draw any conclusion on the issue as to which stimulation target is better for the MDS patients. Third, it is important to define the relative locations of the DBS electrodes to the stimulation targets and the activated volume of tissue, which will be of great benefit to start postoperative programming, assess the clinical effects and explain the stimulation-related adverse effects. However, because of absence of available software to achieve the above goals, we can only know the coordinates of DBS electrodes tips relative to the midcommissural point based on the coregistration with pre- and post-operative images in Medtronic StealthStation^®^ surgical navigation station. Finally, the follow-up period in P2 was relatively short. As found in P1, who experienced initial improvement and subsequent worsening in the neurological function after DBS surgery, the long follow-up period is better to fully assess the effects of DBS on the MDS.

In conclusion, our results have indicated DBS may be an effective therapeutic option for MDS with double mutations in DYT1 and DYT11. Moreover, GPi DBS may be used as a supplementary treatment when initial Vim DBS fails to control MDS symptoms. However, randomized controlled trials between pallidal and thalamic stimulation are needed to determined which site is optimal for DBS in MDS.

## Patients and Methods

Two patients with refractory MDS from different families in different regions were treated with bilateral DBS between 2011 and 2015 at Department of Functional Neurosurgery, Xuanwu Hospital, Capital Medical University, China. The diagnosis of MDS was established according to published criteria^3^,^49^. Genetic testing for DYT1 and DYT11 was performed through polymerase chain reaction amplification and direct sequencing of the specific exons in two patients. Demographic and clinical data were collected as well as details on surgical technique, stimulation parameters and adverse events. Informed consent was obtained from all patients and this study was approved by the Xuanwu Hospital affiliated to Capital Medical University. In addition, all the procedures in this study was in accordance with the approved guidelines.

### GPi/Vim-DBS procedures

Surgery was performed according to established protocol of DBS procedures in our center. Under local anesthesia, the implantation of the DBS electrodes was performed bilaterally using a CRW stereotactic frame (Radionics, Burlington, MA, USA) and MRI-guided targeting with StealthStation^®^ surgical navigation system (Medtronic, Minneapolis, MN, USA). The GPi target was set at 18–22 mm lateral, 4–6 mm inferior and 2–3 mm anterior to the midcommissural point while the coordinate for the target of the Vim was: 13–15 mm lateral, 0 mm inferior and 5–6 mm anterior to the posterior commissural point. These coordinates defined the base of the posteroventral GPi just above the optic tract based on the Schaltenbrand-Wahren anatomical atlas. Single-track microelectrode recording using the Alpha Omega system (Alpha Omega Engineering, Nazareth, Israel) was performed, and neuronal activity was recorded starting from 10 mm above the GPi/Vim target. Surface electromyogram (EMG) activity was recorded before surgery and in the operating room at the same time as neuronal recording. After the initial localization of the target point, the DBS electrodes (Medtronic 3387 in GPi DBS or 3389 in Vim DBS) with four contact points were positioned. Furthermore, during subsequent macro-stimulation after initial implantation to investigate the threshold for the side effects, minimal adjustments might be made to verify the optimal lead location. Finally, the DBS electrodes were connected to an implantable pulse generator (Kinetra, Activa^®^ RC or Activa^®^ PC, Medtronic) implanted in the subclavicular region while the patient was under general anesthesia.

### Postoperative programming

To relieve the MDS symptoms, we commonly began the DBS programming three days after surgery. During the first programming, the threshold for persistent adverse effects such as visual phosphenes, muscle contractions, or involuntary movements was determined for each electrode contact in unipolar mode by using a frequency of 130 Hz and a pulse width of 60 μs. And then the maximal clinical effects of unipolar stimulation with each electrode were determined individually. The active therapeutic contacts were selected when clinical benefit, such as improvement of myoclonus, was maintained at the lowest parameters. The DBS setting would be adjusted according to the patients’ response during the follow-up visit to achieve optimal control of dystonia and myoclonus without side effects.

### Clinical outcome

To quantify the effects of DBS on the clinical outcomes, myoclonus and dystonia were assessed before and after surgery. Myoclonus was assessed using the UMRS rest and action subscores (0–288). The movement subscore (0–120) of the BFMDRS was used to rate the severity of dystonia while the disability subscore (0–30) of the BFMDRS was used to evaluate functional impairment and quality of life.

## Additional Information

**How to cite this article**: Wang, J.-W. *et al*. Deep brain stimulation for myoclonus-dystonia syndrome with double mutations in DYT1 and DYT11. *Sci. Rep.* **7**, 41042; doi: 10.1038/srep41042 (2017).

**Publisher's note:** Springer Nature remains neutral with regard to jurisdictional claims in published maps and institutional affiliations.

## Figures and Tables

**Table 1:** Demographics and medical treatment in the two MDS patients.

	Sex (M/F)	Age at surgery (years)	Age at onset (yeas)	DYT1	DYT11	Predominant complaints on admission	Myoclonus distribution	Dystonia distribution	Alcohol sensitivity	DBS	Previous medical treatment	Follow up (months)
P1	M	30	12	c.904_906delGAG, p.Glu303del(exon5)	c.1294 A > C, p.Ser432Arg (exon10)	myoclonus	neck, trunk, UL, LL	voice, neck, trunk, UL, LL	+	Vim, GPi*	trihexyphenidyl, selegiline, clonazepam	54
P2	M	17	11	c.904_906delGAG, p.Glu303del(exon5)	c.1294 A > C, p.Ser432Arg (exon10)	hand dystonia	UL, LL	voice, UL, LL	Not test	Vim	clonazepam	6

MDS: myoclonus dystonia syndrome, M/F: Male/Female, DYT1: Torsin A gene mutation, DYT11: SGCE gene mutation, +: positive, −: negative, GPi: globus pallidus internus, Vim: ventral intermediate thalamic nucleus, UL: upper limb, LL: lower limb. *Initial bilateral Vim DBS was performed and subsequent bilateral GPi DBS was offered at 43 months after initial surgery.

**Table 2:** DBS programming parameters and coordinates of DBS electrodes tips relative to the midcommissural point.

	DBS targets	Side (R/L)	Active electrodes	Frequency (Hz)	Pulse width (μs)	Voltage (V)	Lateral (mm)	AP (mm)	Vertical (mm)
P1	Vim	R	C + 1-3-	160	90	3.5	18.3	−6.0	−1.4
		L	C + 1-3-	160	90	3.5	−13.8	−9.3	−2.3
	Gpi	R	C + 2-	130	80	3.5	22.0	1.4	−6.4
		L	C + 3-	130	70	2.5	−21.2	2.0	−7.1
P2	Vim	R	C + 0-1-	160	90	3.2	12.4	−5.9	−2.2
		L	C + 1-	160	90	3.4	−12.2	−5.6	−2.6

DBS: deep brain stimulation, AP: anterior-posterior, R/L: right/left.
